# Fibroepithelial Polyp With Actinic Keratosis at Its Base and a Cutaneous Horn Arising From Its Superior Pole: A Clinicodermoscopic-Histopathologic Correlation

**DOI:** 10.7759/cureus.109432

**Published:** 2026-05-22

**Authors:** Chaimae Bouhamdi, Hanane Baybay, Kawtar El Fid, Laila Tahiri Elousrouti, Fatima Zahra Mernissi

**Affiliations:** 1 Department of Dermatology, Hassan II University Hospital, Fez, MAR; 2 Department of Pathology, Hassan II University Hospital, Fez, MAR

**Keywords:** actinic keratosis, cutaneous horn, dermoscopy, fibroepithelial polyp, photodamaged skin

## Abstract

We report an uncommon tripartite topography in a 59-year-old man with chronically photodamaged facial skin, comprising a fibroepithelial polyp with actinic keratosis at its base and a cutaneous horn arising from its superior pole. This case illustrates how unusual structural associations may emerge in actinically damaged skin and broadens the spectrum of horn-bearing lesions. It also underscores the importance of complete excision with integrated clinicodermoscopic-histopathologic correlation for precise characterization and exclusion of invasive carcinoma.

## Introduction

A cutaneous horn (cornu cutaneum) is a conical projection of compact keratin representing a morphologic reaction pattern rather than a specific histologic entity [1,2]. Actinic keratosis is an ultraviolet (UV)-induced intraepidermal keratinocytic atypia arising on chronically photodamaged skin, whereas a fibroepithelial polyp, also termed acrochordon or molluscum pendulum, is a benign pedunculated fibroepithelial proliferation [3,4].

The horn’s clinical significance lies in the underlying base, which may be benign, premalignant, or malignant [1,3]. Across published literature, fibroepithelial polyps have been reported as a miscellaneous benign fibrous horn substrate rather than as a common quantified cause [4-6]. Therefore, the association of a fibroepithelial polyp with actinic keratosis at its base and a cutaneous horn arising from its superior pole appears uncommon. We report a case with clinicodermoscopic-histopathologic correlation illustrating this distinctive tripartite topography.

## Case presentation

A 59-year-old college professor with no comorbidities and Fitzpatrick skin phototype IV was under dermatologic surveillance for chronic facial actinic damage, in the context of lifelong absence of regular photoprotection. He presented with an asymptomatic conical lesion in the infra-palpebral region that had developed and progressively enlarged over two weeks.

**Figure 1:**
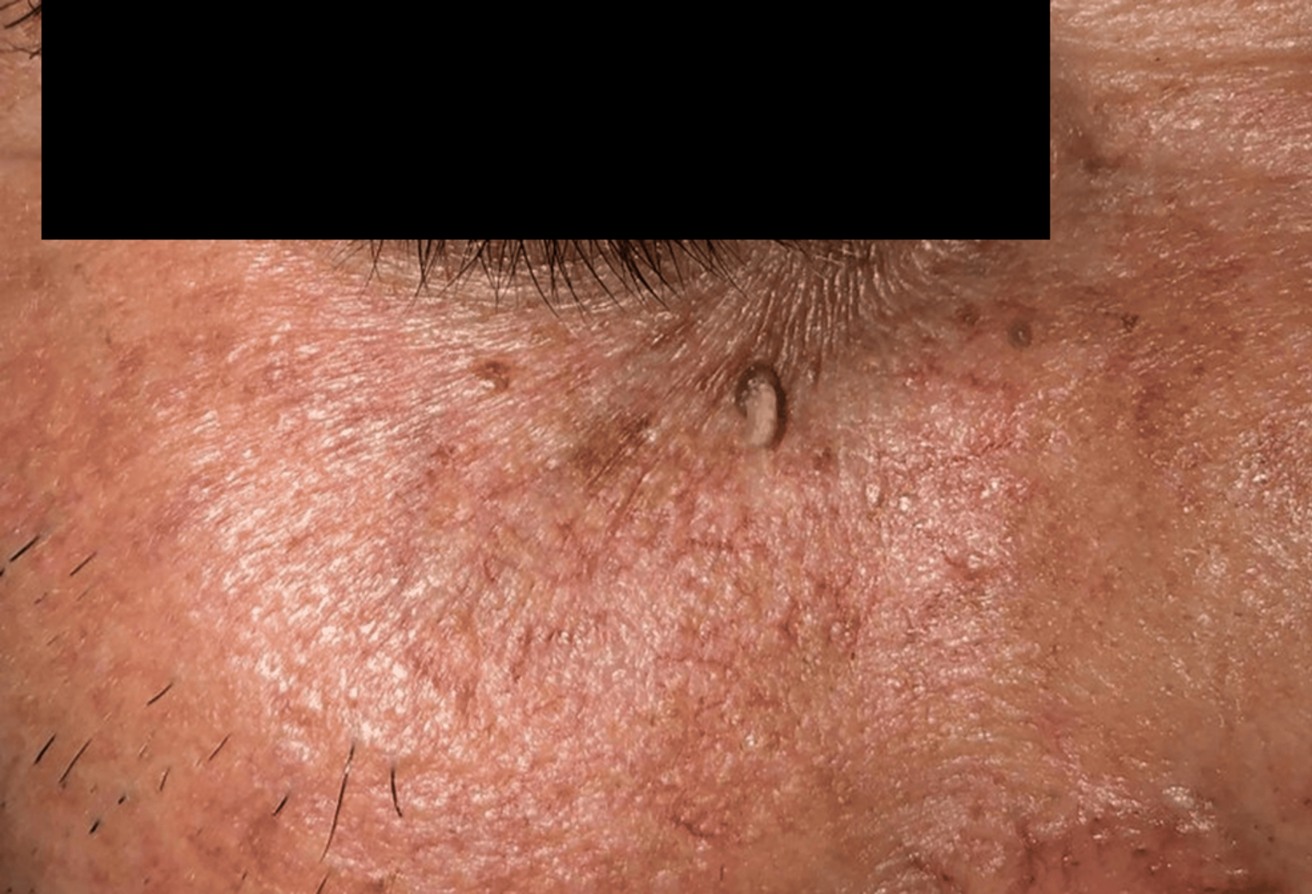
Clinical presentation. Solitary yellow, firm, polypoid cutaneous horn arising from a pigmented pedunculated papule.

Examination revealed a solitary yellow, firm, polypoid cutaneous horn arising from a soft pigmented papule. The base was non-indurated (Figure 1). No regional lymphadenopathy was detected.

**Figure 2:**
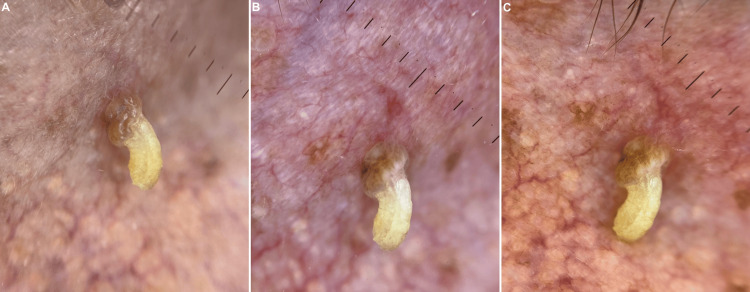
Polarized dermoscopy. (A) Vertically organized horn-substrate-erythematous base complex. (B) The base showed uniform erythema without vascular structures; the surrounding skin displayed chronic actinic damage with telangiectasia and multiple pigmented pseudonetworks. (C) Compact cylindrical keratin column with structureless areas and focal yellow-white-brown concentric lamellar lines, arising from a symmetric brown pedunculated papule with a small pedicle, showing regular brown dots, globules, and fingerprint-like lines, with focal whitish structureless areas at the site of horn emergence.

Polarized dermoscopy showed a compact cylindrical keratin horn, predominantly structureless, with focal yellow, white, and brown concentric lines. The horn measured 4 mm in length and 2 mm in width and arose from a 3 mm brown pedunculated papule with a 1 mm pedicle. The papular component showed regular brown dots and globules, fine fingerprint-like brown lines, and focal whitish structureless areas within the pedicle and at the point of horn emergence. The underlying base displayed uniform erythema without any specific dermoscopic pattern or discernible vascular structures. The surrounding skin showed solar lentigines and telangiectasias, consistent with chronic actinic damage (Figure 2).

**Figure 3:**
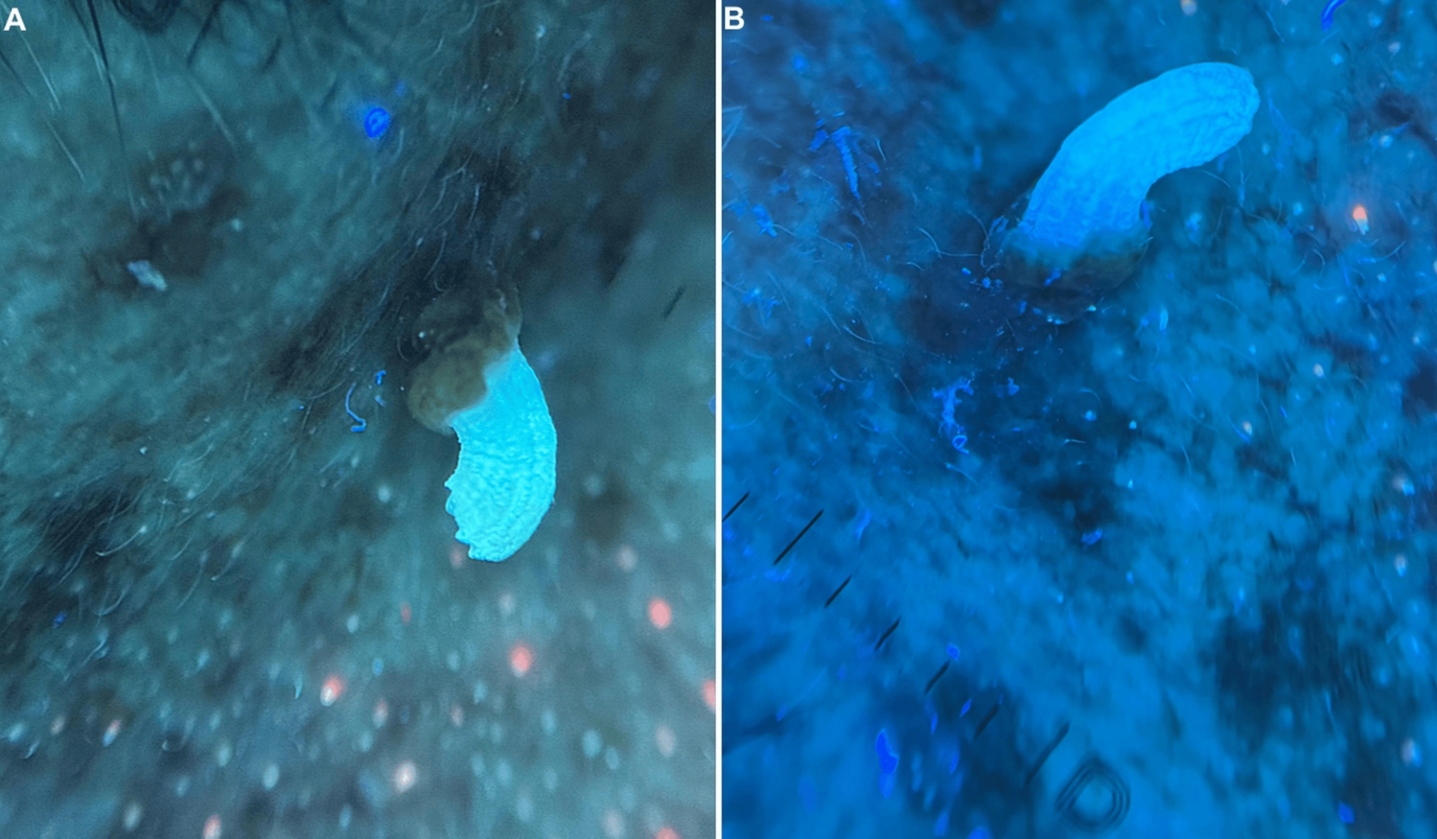
Ultraviolet dermoscopy. (A, B) White-bluish cylindrical keratin column with compact concentric lamellar architecture, sharply demarcated from a symmetric pigmented pedunculated substrate with regular dots and globules and a darker pedicle on a darker base.

UV dermoscopy displayed a white-bluish cylindrical keratin column with compact white and pigmented concentric lines, sharply emerging from a symmetric pigmented pedunculated substrate with regular brown dots and globules and a dark pedicle on a darker base (Figure 3).

Overall, the clinicodermoscopic appearance favored a cutaneous horn arising from a fibroepithelial polyp on a non-specific erythematous base. In the context of chronically photodamaged facial skin, this base raised concern for an underlying keratinocytic lesion, mainly actinic keratosis or squamous cell carcinoma, in situ or invasive.

**Figure 4:**
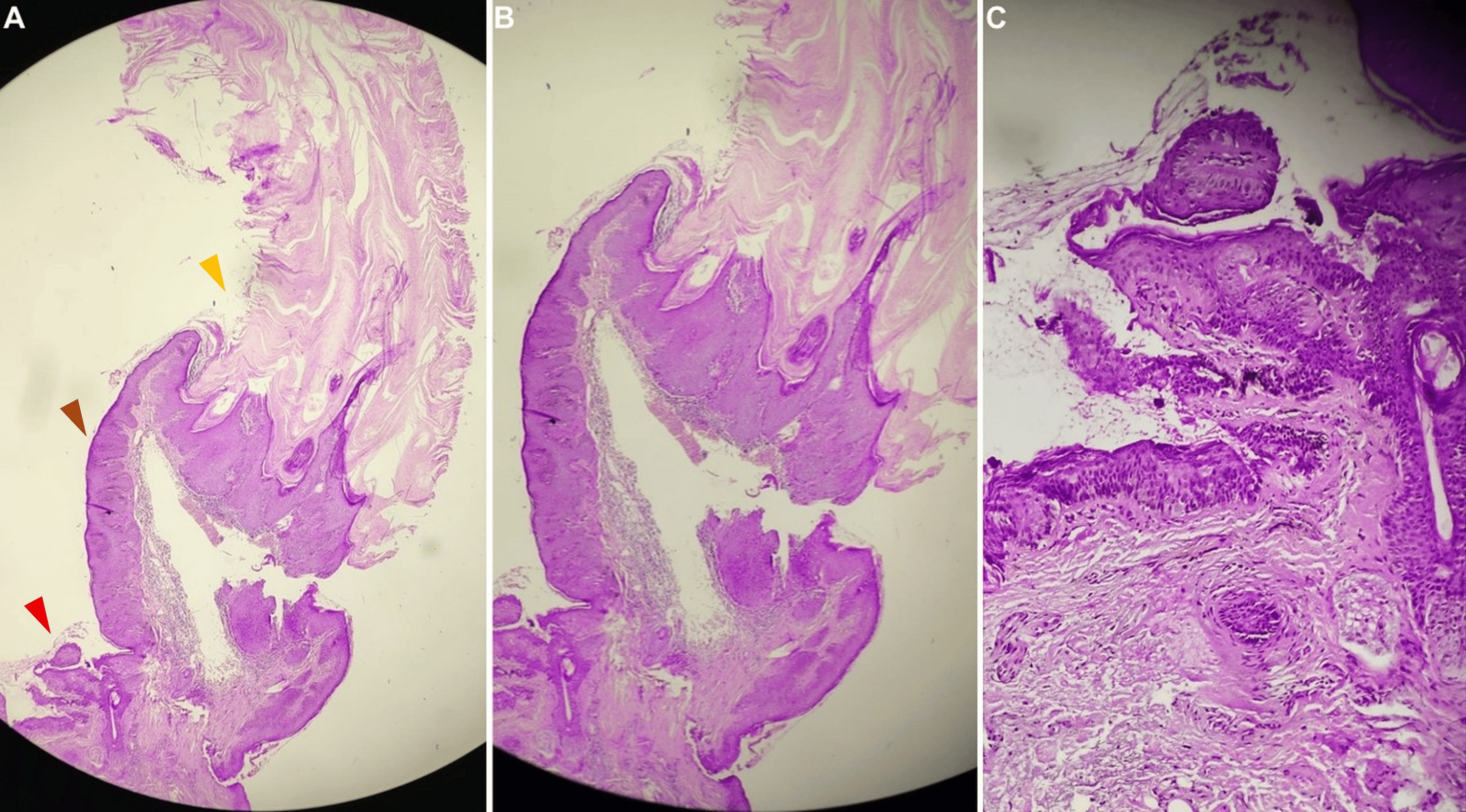
Histopathologic findings. (A) Cutaneous horn (yellow arrowhead). Fibroepithelial polyp (brown arrowhead). Actinic keratosis (red arrowhead). (A, B) Cutaneous tissue of a polypoid architecture lined by hyperplastic stratified squamous epithelium, supported by a fibrovascular core containing sparse lymphocytic inflammatory cells and mild vascular congestion, surmounted by a corneiform orthokeratotic hyperkeratosis. The epidermis at the base of the polyp showed architectural disorganization with keratinocytic atypia and occasional mitoses involving basal and suprabasal keratinocytes. The dermis showed solar elastosis with a mild perivascular lymphocytic inflammatory infiltrate and no tumoral infiltration (hematoxylin, eosin, and saffron (HES), ×40; ×100). (C) Higher magnification highlighting actinic keratosis at the base of the fibroepithelial polyp (HES, ×400).

Histopathologic examination of the completely excised lesion, including its base, showed a polypoid cutaneous lesion lined by hyperplastic stratified squamous epithelium and supported by a fibrovascular core containing sparse lymphocytic inflammatory cells and mild vascular congestion. The lesion was surmounted by a superficial corneiform orthokeratotic hyperkeratosis. The epidermis at the base of the polyp showed architectural disorganization with keratinocytic atypia and occasional mitoses involving basal and suprabasal keratinocytes. The dermis showed solar elastosis with a mild perivascular lymphocytic inflammatory infiltrate and no tumoral infiltration (Figure 4).

Taken together, the lesion consisted of a fibroepithelial polyp covered by epidermis. The epidermis at its base showed actinic keratosis, whereas a cutaneous horn arose from the epidermis covering its superior pole. No histopathologic features of squamous cell carcinoma, either in situ or invasive, were identified. At three-month follow-up, the excision site had healed without clinical or dermoscopic recurrence, and the patient remained under dermatologic surveillance for chronic actinic damage.

## Discussion

A cutaneous horn is clinically striking but diagnostically non-specific. It represents a morphologic reaction pattern to an underlying epidermal lesion, and its clinical significance is determined by the basal pathology, which may be benign, premalignant, or malignant [1,2,5,7]. Across the literature, the most frequent horn-associated substrates are actinic keratosis, squamous cell carcinoma, seborrheic keratosis, and verruca vulgaris [6]. Dermoscopy may assist risk stratification by assessing the horn-to-base relationship, terrace morphology, and base erythema. Horns associated with invasive squamous cell carcinoma more often show a height-to-base diameter ratio < 1, absence of terrace morphology, and presence of base erythema [8].

In our case, the clinically and dermoscopically benign-appearing pedunculated substrate favored a fibroepithelial polyp, a benign fibroepithelial proliferation composed of a fibrovascular core covered by acanthotic epidermis [4]. Dermoscopy showed symmetric pedunculated architecture, a regular pigment pattern, and no atypical vessels or destructive features. However, the erythematous base in chronically photodamaged facial skin raised concern for an underlying keratinocytic lesion, particularly actinic keratosis or squamous cell carcinoma [3,9].

Actinic keratosis may show variant- and grade-dependent dermoscopic patterns, including the strawberry pattern in non-pigmented facial actinic keratosis, pigment-related structures in pigmented actinic keratosis, and compact keratin masses in hyperkeratotic/Olsen grade III lesions [3,9]. In the present case, the basal actinic keratosis was clinically and dermoscopically occult, with only non-specific erythema. Its characteristic features may have been obscured by incorporation into a horn-bearing structure. Complete excision including the base was therefore necessary to avoid superficial sampling and establish the definitive diagnosis [5,7,10].

Histopathology established an uncommon tripartite topography with three components in continuity: a fibroepithelial polyp forming the pedunculated scaffold, actinic keratosis located at its base, and a superficial corneiform orthokeratotic horn arising from its superior pole. No histopathologic features of squamous cell carcinoma, either in situ or invasive, were identified. This configuration is best interpreted as the coexistence of a fibroepithelial polyp and actinic keratosis within a chronically photodamaged field, with secondary horn formation, rather than pathogenetic transformation of one component into another. Complete excision was both diagnostic and therapeutic, and continued surveillance with strict photoprotection was indicated because of the underlying actinic field [3].

## Conclusions

This case documents an unusual tripartite horn-bearing lesion, composed of a fibroepithelial polyp, actinic keratosis at its base, and a cutaneous horn arising from its superior pole. The key lesson is that neither the keratin horn nor the benign-appearing pedunculated component determines the diagnosis; rather, definitive diagnosis rests on histopathologic examination of the entire excised lesion, including the base, and correlation with the clinicodermoscopic findings.
